# Roles, responsibilities and characteristics of lay community health workers involved in diabetes prevention programmes: A systematic review

**DOI:** 10.1371/journal.pone.0189069

**Published:** 2017-12-07

**Authors:** Jillian Hill, Nasheeta Peer, Brian Oldenburg, Andre Pascale Kengne

**Affiliations:** 1 Non-Communicable Diseases Research Unit, South African Medical Research Council, Cape Town, South Africa; 2 Department of Medicine, University of Cape Town, Cape Town, South Africa; 3 Melbourne School of Public Health and Global Health, University of Melbourne, Melbourne, Australia; University of Tennessee Health Science Center, UNITED STATES

## Abstract

**Aim:**

To examine the characteristics of community health workers (CHWs) involved in diabetes prevention programmes (DPPs) and their contributions to expected outcomes.

**Methods:**

Electronic databases including PubMed-MEDLINE, *EBSCOHost*, and SCOPUS/EMBASE were searched for studies published between January 2000 and March 2016. All studies that used CHWs to implement DPP in ≥18-year-old participants without diabetes but at high risk for developing the condition, irrespective of the study design, setting or outcomes measured, were included. Results were synthesized narratively.

**Results:**

Forty papers of 30 studies were identified. Studies were mainly community-based and conducted in minority populations in USA. Sample sizes ranged from 20 participants in a single community to 2369 participants in 46 communities. Although CHWs were generally from the local community, their qualifications, work experience and training received differed across studies. Overall the training was culturally sensitive and/or appropriate, covering topics such as the importance of good nutrition and the benefits of increased physical activity, communication and leadership. CHWs delivered a variety of interventions and also screened or recruited participants. The shared culture and language between CHWs and participants likely contributed to better programme implementation and successful outcomes.

**Conclusions:**

The complexity of DPPs and the diverse CHW roles preclude attributing specific outcomes to CHW involvement. Nevertheless, documenting potential CHW roles and the relevant training required may optimise CHW contributions and facilitate their involvement in DPPs in the future.

## Introduction

The rapid worldwide increase in type 2 diabetes (henceforth referred to as diabetes) has led to the development of a variety of different delivery models to prevent the development of this condition. To this end, a number of large randomised controlled trials (RCT) have demonstrated that lifestyle interventions reduce the incidence of diabetes between 29% and 58% in high-risk populations, and this can be maintained for well over 10 years [1]. However, these programmes when conducted under research conditions are usually resource intensive and thus, not practical or feasible to conduct in primary healthcare (PHC) or community-based settings. Particularly costly is employing professional healthcare workers to implement such interventions, it is not the best use of this resource, especially in developing regions with shortages of skilled healthcare workers.

The global need for efficient and cost-effective use of healthcare resources, particularly in low-income countries has led to the introduction of lay health workers or non-professional community health workers (CHWs) to fulfil a variety of tasks and roles such as patient care, education, support for care delivery, care coordination and social support, especially in remote areas or among minority groups [2, 3]. CHWs have become involved with supporting and filling a variety of capacities in healthcare programmes [4], including in relation to diabetes prevention programmes (DPPs). CHW-led support services to improve health outcomes range from small community-based initiatives to large national programmes. CHWs can be an important link between the community and healthcare, an intervention programme or service delivery by providing context-specific support [2, 5], which can lead to better long-term outcomes for the participants. Such individuals usually have commonality with the community they serve in terms of ethnicity, language, socioeconomic status and life experiences [6]. Rosenthal et al. [7] refers to CHWs as an umbrella term which includes “outreach workers, *promotores (as) de salud*, community health representatives, and patient navigators”. In a systematic review and meta-analysis of 28 United States (US)-based DPPs, Ali and colleagues [8] described the successes of CHW implemented DPPs. Most significantly, they reported that CHW-delivered interventions were associated with a similar change in weight at follow-up compared with health professional implemented programs [8].

To improve the delivery and outcomes of DPPs that CHW’s are involved with, it is important to identify the key characteristics of CHWs as well as the required training and level of support that are required. However, there is a shortage of such comprehensive assessments in the literature. Therefore, this systematic review aims to examine CHW implemented DPPs and describe the key characteristics that contributed to positive outcomes.

## Methods

### Data sources

We developed a systematic review protocol using guidelines described by the Preferred Reporting Items for Systematic review and Meta-Analysis Protocols (PRISMA-P) [9], which was registered on the PROSPERO Register (CRD42016043237). The electronic databases searches were done on MEDLINE via PubMed Central, *EBSCOHost*, and SCOPUS/EMBASE. Free text as well as Medical Subject Headings (MESH) were used, including community health workers, lay health workers, non-professional health workers, *Promotores de Salud*, community health aids, peer advisors, community health advisors, village aids, community aids, lay counsellors, health promotores, community health promotores, village health volunteers, lay health educators diabetes, diabetes mellitus, type 2 diabetes, and prevention. Boolean operators, such as AND/OR/NOT were used to string terms together. Searches were limited to publications in English. For example in PubMed Central the search strategy was the following:

("community health workers"[MeSH Terms] OR ("community"[All Fields] AND "health"[All Fields] AND "workers"[All Fields]) OR "community health workers"[All Fields]) AND ("diabetes mellitus"[MeSH Terms] OR ("diabetes"[All Fields] AND "mellitus"[All Fields]) OR "diabetes mellitus"[All Fields] OR "diabetes"[All Fields] OR "diabetes insipidus"[MeSH Terms] OR ("diabetes"[All Fields] AND "insipidus"[All Fields]) OR "diabetes insipidus"[All Fields]) AND ("prevention and control"[Subheading] OR ("prevention"[All Fields] AND "control"[All Fields]) OR "prevention and control"[All Fields] OR "prevention"[All Fields])

Additional methods to identify studies included manually searching journals and conference proceedings, checking reference lists, and identifying unpublished data. One author and an independent assessor (JH, LM) independently identified potentially relevant studies by reviewing titles and abstracts retrieved from the aforementioned databases. The full texts of studies identified as potentially relevant were retrieved and screened in duplicate for inclusion. Consensus was achieved through discussion and, when needed, consultation with a senior author (APK).

### Definition of community health workers

For the purpose of this review, a CHW is any lay or non-professional health worker involved with the delivery of a diabetes prevention programme, either as a volunteer or for a stipend. This accords with Norris’s description of a CHW as an individual without formal healthcare training but trained to deliver context-specific healthcare to a community with whom s/he has a relationship [2].

### Study inclusion criteria

All studies that used CHWs to implement DPP in ≥18-year-old participants without diabetes but at high risk for developing the condition, irrespective of the study design, setting or outcomes measured, were included. Studies that examined outcomes only among the CHWs, e.g. reports of CHW training interventions were also included because the aim of this systematic review is to establish the key characteristics of CHWs who contribute to successful DPP.

Eligible studies included those published in peer-reviewed journals from January 2000 until March 2016. Studies were excluded if they: 1) focused on diabetes management, 2) did not make use of CHWs, 3) comprised interventions of less than three months’ duration, or 4) were narrative reviews, opinion pieces, letters to the editor or any other form of publication without primary data.

### Data extraction and synthesis

Two data extraction tables summarise the data from the included studies. Table 1 shows the study-specific details such as the authors’ names, demographic data, methodology and outcomes. Table 2 describes the CHW-specific characteristics including gender, age, education level, the training and support they received, and their role in the intervention. JH entered the summary data in the tables and LM then checked these. Given that the studies included in this systematic review were not sufficiently similar for a meta-analysis, data were synthesised narratively. We compiled thematic summaries relevant to the review objective and research questions stated in the systematic review protocol (unpublished).

**Table 1:** Study characteristics.

Author; Year published	Country/Region; Setting	Study design, intervention and comparator	Participant characteristics	Outcomes measure	Effect of intervention	Length of intervention
1. West et al., 2011 [10] 2. Krukowski et al., 2013 [11] 3. Krukowski et al., 2013 [12]	Arkansas—USA; Senior centres	**Design:** Cluster RCT [lifestyle weight loss vs. cognitive training programme]	N = 228 Gender: 84% Female Age range: 60+ Race/ethnicity: 92% white	Primary: Weight loss of ≥5% Secondary: Examination of costs Tertiary: Evidence to train lay health educators (LHE) to implement weight loss interventions	Physiological outcome: 3.7 kg weight loss at 4 months Cost/economic outcome: a LHE-delivered DPP translation is effective in achieving weight loss at low cost at senior centres Implications for training LHE: Methods used produced favourable long-term retention of LHE’s and good adherence to intervention protocol	-12 weekly group sessions -60 minutes each
4. Perez et al., 2015 [13]	Philadelphia (US); clinical and community settings	**Design:** RCT–parallel group design [standard care vs. metformin/ DDP lifestyle intervention to normal care] - 3-arm RCT	N = 92 Gender: 100% Female Age range: 45.1±12.5 Mean age: ≥20 years Race/ethnicity: Latina	Primary: 12-month weight difference between groups Secondary: cardio metabolic markers	Only baseline characteristics available	24 sessions over 12 months, 12 weekly core sessions followed by ten maintenance sessions [90-minute sessions]
5. Katula et al., 2010 [14] 6. Blackwell et al., 2011 [15] 7. Katula et al., 2011 [16] 8. Katula et al., 2013 [17] 9. Lawlor et al., 2013 [18] [HELP-PD]	Forsythe County, North Carolina (US); Community-based	**Design:** RCT -DPP lifestyle weight loss vs. enhanced usual care	N = 301 overweight & obese volunteers/ Pre-diabetics Gender: 57.5% female Age: ≥ 21 years Mean Age: years: 57.9 ±9.5 Race/ethnicity: mostly white (73.8%)	Primary: change in fasting glucose Secondary: change in CVD risk factors, health-related quality of life & social cognitive variables Tertiary: cost effectiveness	**1 year results**: Physiological outcomes: Intervention participants experienced significantly greater decreases in: -blood glucose (-4.3 vs. -0.4mg/dL; p <0.001) -insulin (-6.5 vs. 2.7 μU/mL; p <0.001) -homeostasis model assessment of insulin resistance (-1.9 vs. 0.8; p <0.001) -weight (-7.1 vs. -1.4 kg; p <0.001) -BMI (-2.1 vs. -0.3 kg/m^2^; p <0.001) -waist circumference (-5.9 vs. -0.8 cm; p <0.001) **2 year results:** Physiological outcomes: -LWL participants significantly greater decreases in fasting glucose (-4.35 mg/Dl); insulin (-3.01 μU/ml); insulin resistance (-0.97); body weight (-4.19 kg); waist circumference (-3.23 cm); BMI (-1.40), with all p-values <0.01. Cost/economic outcome: Substantially lower medical costs.	24 months [weekly sessions for 6 months, followed by monthly sessions (7–24 months]
10. Islam et al., 2013 [19]	New York [Korean Community], Community setting	**Design:** RCT	N = 48–25 in treatment group & 23 in control group Gender: 64% female Age: 18–75 years Mean Age: 59.7 years Race/ethnicity: Korean	Primary: clinical measurements Secondary: health access	No significant differences between treatment & control groups Changes in: Physiological Outcomes: -weight, -waist circumference,—systolic blood pressure, -physical activity Behavioural outcomes: -nutrition and diabetes knowledge as well as mental health	6 month intervention period
11. Horowitz et al., 2011 [20] [Project HEED]	East Harlem, New York (US); Community-based	**Design:** RCT: Using a participatory approach, the partnership chose to focus on diabetes prevention, & co-developed all intervention, recruitment, research & evaluation strategies	N = 99 Gender: 85% female Age: ≤ 18 Race/ethnicity: 87% Latino	Primary: effectively translating diabetes prevention Secondary: weight loss/impact local health	Physiological Outcome: Achieved statistically significant weight loss	8 sessions
12. Duggan et al., 2014 [21]	Yakima Valley (rural area) (US); Educational sessions conducted in participants’ homes	**Design:** A parallel two-arm RCT	N = 228 Gender: 70.6% female Mean Age: 50.6 years Race/ethnicity: Hispanics	Primary: HbA1c levels Secondary: Fruit & vegetable consumption Tertiary: Frequency and intensity of physical activity	Physiological Outcome: -Intervention group showed significant improvement in HbA1c scores (-37.5%, p = 0.4) Behavioural Outcome: -a trend of greater increases in the frequency of moderate & vigorous physical activity in the intervention group	5 weeks
13. Koniak-Griffin et al., 2015 [22]	Southern California (US); Community settings	**Design:** A community prevention model was employed in planning & implementing this RCT	N = 223 Gender: Females Age: 35–64 years Mean age: 44.6 years Race/ethnicity: Latina	Primary: Evaluate the effects of a lifestyle behaviour intervention by specially trained promotoras Secondary: Acceptability & feasibility of intervention	Physiological Outcomes: -Statistically significant positive effect on waist circumference -Receiving higher intensity (dosage) of the intervention was modestly beneficial in terms of greater improvement in body mass index, weight, and waist circumference. Behavioural Outcomes: - Statistically significant positive effect on dietary habits, patterns of physical activity (daily steps), and knowledge about heart disease (e.g., risk factors, prevention measures).	6 month lifestyle behaviour intervention
14. Sathish et al., 2013 [23]	Rural Kerala, India; Community-based	**Design:** A cluster RCT of a peer-led lifestyle intervention	Inclusion criteria: Gender: Males & females on the electoral roll Age: 30–60 years Race/ethnicity: Malayan	[EXPECTED] Outcomes: Behavioural outcomes -improved diet -increased physical activity -reduced tobacco use -reduced alcohol consumption Psychosocial outcomes -reduced stress -improved quality of life Clinical outcomes -reduced BP -reduced waist circumference -reduced body fat Biochemical outcomes -reduced incidence of diabetes -improved glycaemic control -improved lipid profile		-11 peer-led small group sessions -2 education sessions led by experts
15. Simmons et al., 2008 [24]	Te Wai o Rona (New Zealand); Community-based	**Design:** A cluster-RCT of intensive lifestyle change [pilot study cohort]	N = 212 Vanguard: Gender: 34.4% male Mean age: 47 years Control: Mean age: 50 years Gender: 40.4% male Race/ethnicity: Maori	Primary: 35% reduction in incident diabetes among Maori Secondary: cost effectiveness	Physiological Outcome: -significant weight loss among all participants in pilot (-1.3 (SD 3.6) kg, p <0.001) Intervention Acceptability: -Participants & CHW found the messages, toolkit & delivery approach acceptable	-not clear -mean of 189 days of intervention over study period
16. DeJoy et al., 2013 [25] [FUEL Your Life]	Union Pacific Railroad locomotive maintenance facility (US); Worksite	**Design:** **Intervention development** **Formative:** stakeholder interviews, focus groups, and observation of work environment **Pilot study method:** single group design with data collection at three time points: baseline, 6 months, and 12 months	N = 167 Gender:97% men Age: 20-69years Mean age: 47 years Race/ethnicity: 85% white	Primary [pilot study]: Adaption/translation of DPP for the workplace Secondary: weight loss	Physiological Outcome: -Modest but statistically significant weight reductions at both 6 (core intervention period) and 12 months (maintenance period)	24 weeks
17. Brace et al., 2015 [26] [FUEL Your Life]	Union Pacific Railroad locomotive maintenance facilities (US); Worksite	**Intervention Evaluation:** The RE-AIM (Reach Effectiveness Adoption Implementation Maintenance) framework was used to examine the factors that influenced programme implementation using data from an environmental assessment, participant surveys, peer health coach surveys, peer health coach surveys, and occupational health nurse surveys	N = 479 employees enrolled Gender: 94% male Mean age: 46 years Race/ethnicity: 75% Caucasian [236 participants at post-test]	Primary outcomes: -7% weight loss -150 minutes of physical activity Secondary: Process evaluation	Physiological Outcome: Overall, the programme was effective for weight maintenance.	Duration of FUEL Your Life
18. Wilson et al., 2016 [27] [FUEL Your Life]	Union Pacific Railroad locomotive maintenance facilities (US); Worksite	**Intervention implementation:** RCT–five worksites	N = 362 workers Gender: 94% male Mean age 46 Race/ethnicity: predominantly white (80.6% control, 71.60% treatment)	Primary outcomes: -7% weight loss -150 minutes of physical activity	Physiological Outcome: Participants in the intervention group maintained weight/BMI (-.1 pounds/-.1 BMI), whereas the control participants gained weight/BMI (+2.6 pounds/+.3 BMI), resulting in a statistically significant difference between groups. Fifty-five percent of intervention participants lost some weight, whereas only 35% of the control group lost weight	12 months
19. Ockene et al., 2012 [28]	Lawrence, Massachusetts (US); Individual sessions at home & group sessions at senior centre (community site)	**Design:** Lifestyle intervention care vs. Usual care	N = 312 Gender:74% female Age: 25–79 years Mean age: 52 years. Race/ethnicity: Hispanic/Latino	Primary: weight loss & diabetes risk reduction	Physiological Outcomes: -Intervention group had a moderate but significant weight reduction (-2.5 vs. 0.63lb; p = .04) -clinically meaningful reduction in HBA1c (-0.10% vs. -0.04%; p = .009) -improved insulin resistance	-Three individual and 13 group sessions over a 12-month period
20. Staten et al., 2012 [29] [Pasos Adelante/ “Steps Forward”]	Douglas Arizona (US-Mexico border); Community setting	**Design:** Quasi experimental pre-test, post-test study with follow-up	N = 305 (conclusion of programme 254, 3-month follow-up 221) Gender: 92% female Mean Age: 54.4 years Race/ethnicity: Hispanic	Primary: Improvements in select physiological measures	Physiological Outcomes: Decreases in: - BMI (p = 0.04) -waist & hip circumference (p <0.001) -diastolic & systolic BP (p <0.001) -total cholesterol (p = 0.008) -glucose levels improved between conclusion & follow-up (p = 0.01)	12 week sessions
21. Islam et al., 2014 [30] [Project RICE]	New York (US); Community-based	**Design:** A quasi-experimental two-arm design pilot study	N = 126 baseline (108 completed baseline & 6-month follow-up) Gender: 96% female (treatment grp.) Age: 18–75 years Mean age: 46.3 years (treatment grp.) Race/ethnicity: Sikh Asian Indian immigrants	Primary: clinical variables (weight, BMI, waist, BP, glucose & cholesterol) Secondary: health behaviours	Physiological Outcomes: Significant changes for treatment group in: -weight BMI -waist circumference -BP -glucose -physical activity -food behaviours -diabetes knowledge	6-month intervention period
22. Thompson et al., 2015 [31]	Rural Virginia (US); Housing complexes/camp	**Design:** Pilot study (quasi-experimental)	N = 66 [Migrant farmworkers] Gender: Males Age: 18–64 years Mean age: 33.67 years Race/ethnicity: Mexican	Primary: Latino CHW can use non-invasive diabetes & CVD screening tools with similar accuracy as a registered nurse	-CHW perform similarly to registered nurses in the use of non-invasive DM and CVD tools -Risk screening	One screening session. One follow-up call, if required
23. Oba et al., 2011 [32]	Thailand; Community setting	**Design:** Three-stage approach -situational analysis -model development -testing	N- = 160 Gender: 91% female Age: > 35 years Mean age: 45.06 years Race/ethnicity: Thai [Pre-diabetics]	Primary: to promote positive behaviour & lifestyle changes Secondary: to evaluate the effectiveness of the programme	Physiological Outcomes: -BMI score lower (p = 0.001) -waist circumference lower (p = 0.01) -systolic BP lower (p = 0.01) Behavioural Outcome: -Mean score for physical activity increased (p≤0.01)	+3 times a week for 3 months
24. Balagopal et al., 2012 [33]	Gujarat (India); Community setting (rural villages)	**Design:** A community-based participatory research method used to plan & tailor the intervention by engaging trained CHW as change agents to provide lifestyle education, serve as community advocates & collect data	N = 1638 Gender: 53% female Age: ≥ 18 years Mean age: 41.9 years Race/ethnicity: [rural] Indians	Primary: to evaluate the effectiveness of a CBPR approach to diabetes prevention	Physiological Outcomes: –Reduced blood glucose levels by 5.7 & 14.9 mg/dL for individuals with prediabetes & diabetes respectively -lowered systolic & diastolic BP by 8mm Hg & 4mm Hg respectively in overall population -general & abdominal obesity also decreased by ≤1% Knowledge Improvement: -knowledge of diabetes & CVD improved by 50% in the high SES group & doubled in the low SES group	6 months -lifestyle intervention = five one-on-one and five group based encounters
25. O’Brien et al.,2015 [34]	Philadelphia (US); Community-based	**Design:** A pilot trial	N = 20 Gender: Females Age: ≥ 20 years Mean age: 44.5 years Race/ethnicity: Latina	Primary: to evaluate weight changes & cardio metabolic markers (waist, BP, plasma glucose, insulin, HbA1c, lipids) Secondary: assess the feasibility & acceptability of intervention	Physiological Outcomes: -Mean weight loss of 10.8 pounds at 12 months -significant pre-post reductions in waist circumference, diastolic BP, LDL cholesterol & insulin levels -modest reductions in A1c and fasting plasma	12 months -24 session curriculum
26. Benyshek et al., 2014 [35] [The Life in BALANCE pilot study]	Nevada, Las Vegas (US); Community-based resource centre	**Design:** A pilot translational study modelling the DPP intensive lifestyle coaching intervention	N = 22 [only 12 at the final post-programme follow-up] Gender: 83% female Age: ≥ 21 years Mean age: 39.6 years Race/ethnicity: American Indians / Alaska Natives	Primary: translation of DPP for urban American Indians / Alaska Natives Secondary/Clinical measures: weight, BMI, waist, BP, blood lipids, fasting blood glucose, HbA1c	Physiological Outcomes: -Significant decreases in waist circumference & elevated HDL cholesterol -triglycerides manifested the highest percentage change without statistical significance	16 weeks intensive life coaching intervention
27. Philis-Tsimikas et al., 2014 [36]	San Diego County (US); Community clinic setting	**Design:** A single-group pre-post design was utilised	N = 84 Gender: Females Age: 18–45 years Race/ethnicity: Latina	Primary: HbA1c Secondary: lipids & BP Tertiary: [Self-reported]: physical activity, diet, perceived health, fatalistic beliefs, diabetes-specific cultural beliefs	Significant (p <0.05) improvements in: Physiological Outcomes: -lipids -BP Behavioural Outcomes: -physical activity -dietary fat intake Knowledge Improvements: -fatalistic & cultural diabetes beliefs	-8-week intervention -monthly maintenance & support sessions offered to participants following completion of the primary intervention period
28. Cherrington et al., 2015 [37]	Birmingham, Alabama (US); Community setting	**Design:** Developed a theory-based, promotora-delivered intervention to promote weight loss among immigrants an emerging Latina community in Alabama	N = 22 Gender: Females Mean age: 36.5 years Race/ethnicity: Latina	Primary: to develop & pilot test a theory-based, promotora-delivered, peer support weight loss intervention	Physiological Outcomes: -significant weight loss (mean 2.1 kg, SD 2.6, p <0.001) Behavioural Outcomes: -levels of moderate physical activity increased (p <0.05) -dietary practices improved (p <0.001) -depressive symptoms also improved (p <0.001)	8-week intervention
29. Ruggiero et al., 2011 [38]	Southwest Chicago (US); Community setting	**Design:** A nonrandomized prospective study using a single-group design	N = 69 Gender: 92.8% female Age: 18–65 years Mean age: 37.86 years Race/ethnicity: Hispanic	Primary: Anthropometric measures & Secondary: paper & pencil measures were administered to examine programme outcome	Physiological Outcomes: At 6 months: -20% of the sample achieved a 7% weight loss goal -29% achieved a 5% weight loss goal At 12 months: -16% achieved 7% weight loss goal -30% achieved 5% weight loss goal	1 year programme
30. Treadwell et al., 2010 [39]	Lorain county, Ohio (US); Community-based	**Design:** The save our son’s study is a community-based, culturally responsive 7 gender-specific intervention aimed at reducing obesity & diabetes among a small sample of African American men	N = 42 Gender: Males Race/ethnicity: African American	Primary: feasibility of implanting a group health education & intervention model Secondary: access to & utilisation of health care services & community supportive resources	Physiological Outcomes: -decreased BP, weight, BMI Behavioural Outcomes: -increased levels of exercise & fitness activities Knowledge Improvements: -participants had greater knowledge about strategies for prevention & management of obesity & diabetes	6-week intervention
31. Cadzow et al., 2013 [40] 32. Cadzow et al., 2014 [41]	Buffalo city (US); Community settings	**Design:** The Neighborhood Health Talker project aimed to train & implement cultural health brokers primarily targeting communities of colour to improve diabetes knowledge & diabetes self-management skills [Pre-test/Post-test]	N = 13 CHW Gender: 92% female [12 women, one man] Age: 35–65+ years Race/ethnicity: Black/African American +700 community members) N = 208 (survey participants) 61.8% female Mean age: 33.6 years	Primary: evaluation results of a community-based diabetes education pilot project Secondary: Diabetes resource libraries established	Knowledge Improvements: -The training was successful in increasing trainee knowledge and confidence about diabetes prevention and self-management. Behavioural Outcomes: -Participants not only developed proficiency in discussing diabetes, they also made important lifestyle changes that demonstrated their commitment to the cause and the project Intervention Outcomes: -50 community conversations Eight resource libraries	-5 Community conversations -July-October 2009
33. Staten et al., 2005 [42] [Pasos Adelante/ “Steps Forward”]	Yuma & Santa Cruz counties, Arizona (US); Community-based (Churches, schools, health fairs)	**Design:** A 12-week programme facilitated by CHW	N = 248 (216 completed) Mean age: 49.5 Gender: mostly female Race/ethnicity: Hispanics	Primary: primary prevention of chronic disease	Behavioural Outcomes: -Significant increase in moderate to vigorous walking -shifts in nutritional patterns	12-week programme
34. Sosa et al., 2013 [43]	San Antonio, Texas (US)	**Design:** Recruitment, training & evaluation of intervention fidelity of Promotoras de Salud for obesity and diabetes prevention	N = 5 promotoras Gender: Females Age: 38–54 years Mean age: 46.5 years Race/ethnicity: Mexican American] N = 36 participants	Primary: describe training process Secondary: fidelity of study protocol implementation	Knowledge Improvements: -Post knowledge test scores were high (M = 83.5; SD = 6.4) Intervention Acceptability: -study participants perceived promotoras to be supportive & helpful in assisting them to reach their goals	14 sessions, 90 min. each
35. Kousar et al., 2015 [44]	Melbourne (Aus.); homes of community members or CHVs	**Design:** Strengthening capacity of key community volunteers to support diabetes prevention and self-management	N = 17 Community health volunteers Gender: Females Age: 20–60 years Race/ethnicity: Australian immigrants, fluent in at least one non-English language N = 30 Community members Mean age: 44 years Race/ethnicity: Culturally and linguistically diverse	Primary: effectiveness of diabetes prevention & management model	Knowledge Improvements: -Diabetes knowledge among CHVs increased - Diabetes knowledge among community members increased Intervention Outcome: -CHVs were able to collect anthropometric data & knowledge surveys with greater participation than outreach programmes	Not clear
36. Coppell et al., 2009 [45]	Ngati (New Zealand); Community-based	**Design:** Interrupted time-series prevalence surveys with a process evaluation	[2003] N = 286 Gender: 59% female [2006] N = 235 Gender: 59% female Two age groups: -25-49 years. -50+ years Race/ethnicity: Indigenous Ngati Porou (Maori) community	Primary: reduce the prevalence of insulin resistance	Physiological Outcomes: -Insulin resistance prevalence decreased markedly from 35.5% to 25.4% (p = 0.003)	Ongoing
37. Schulz et al., 2005 [46]	Detroit, Michigan (US); Community-based	**Design:** A case study description & analysis of the Healthy Eating & Exercise to Reduce Diabetes project in the theoretical context of a conceptual model of social determinants of health	A community	Primary: reduce the risk/delay onset of diabetes	Intervention Outcome: Barriers and facilitators to applying a social determinants model identified	2 years
38. Cohen & Ingam 2005 [47] 39. Teufel-Shone, Drummond & Rawiel et al., 2005 [48] 40. Reinschmidt et al., 2010 [49]	U.S.-Mexican border; Community-based	**Design:** Community case studies **Design:** Comparative study	Yuma & Santa Cruz communities Race/ethnicity: Predominantly Hispanic (89.1–95.2%)	Primary: description & analysis of a community-based model for diabetes prevention & control Secondary: building capacity of the communities to work together to establish comprehensive, integrated & sustainable diabetes prevention & control programmes Tertiary: Success of academic-community partnership Primary: development and adaptation of a culturally appropriate family-based diabetes program Primary: demonstration of how interventions are adapted at the intersection of multiple cultural contexts Secondary: comparing and contrasting original adaptations–addressing the tension between fidelity and adaption Tertiary: demonstrating the centrality of adaption of negotiations to community-based participatory and capacity building approaches.	Intervention Outcomes: -Building capacity of the communities to work together to create comprehensive, integrated, & sustainable diabetes prevention & control programmes -the overwhelming success of this academic-community partnership to integrate both research & community perspectives to address the devastating toll of diabetes academic Knowledge improvements: -significant increase in diabetes risk knowledge Behaviour Outcomes: -significant decreases in the frequency of intake in sugar sweetened beverages -trend in exercising five or more times a week for 30 minutes or more Significant increase in participants reporting familial support Study Conclusions: - while socio-cultural are not required because the programme is appropriate for a particular population, adaptions that compliments the goals, objectives and resources of local agencies are important -the complexity of experiences, expertise and strengths of the other contexts pertinent to programme adaption requires consideration by public health practitioners - intervention curricula need to be balanced between fidelity and adaption - curricula that is too scripted, ignores the strengths of promotores or other CHW which are at the centre of this balance -adapting programs to the complexity of context is critical to facilitating local ownership and sustainable program	-NP -12 week programme (10 points of contact)
41. Whittemore et al., 2013 [50]	U.S.; Public housing community centres	**Design:** Mixed-method embedded design	N = 67 Gender: 79% female Age: >21 years Mean age: 40 years Race/ethnicity: 79% non-white	Primary: description of implementation process Secondary: barriers & facilitators to programme identified	Intervention Outcomes: -Home-care nurses were able to implement a DPP programme in public housing communities -nurses & CHW were resourceful & positive about programme implementation -linking existing resources is one approach to disseminating DPPs	-6 months

**Table 2:** CHW-specific characteristics.

Authors	Demographics of CHW	CHW eligibility	Training [type] received	Length of training received	Support provided to CHW	Role of the CHW
1. West et al., 2011 [10] 2. Krukowski et al., 2013 [11] 3. Krukowski et al., 2013 [12]	Gender: 90% women Mean age: 61 years Mean education level: 70% reported some college or a college degree	NP	Content: - All aspects of delivering the lifestyle programme - key elements of a behavioural weight-control approach - protocol fidelity Intervention goals -behavioural strategies -giving feedback on self-monitoring diaries -recruitment methods -techniques for conducting effective group sessions -individual session materials reviewed and rehearsed -responsible conduct of research -obtained human subjects protection certification Format: - face-to-face training -experiential learning exercises	32 hours	A weekly technical support conference call	Lay health educators delivered 12 weekly sessions
4. Perez et al., 2015 [13]	Gender: NP Mean age: NP Mean education level: NP	-High school completion -Spanish language fluency -natural leadership skills -commitment to serving their community	Content and Format: -18-hour training on Group Lifestyle Balance protocol -2-day training on diabetes prevention -post-intervention feedback sessions: 50h (2h per session) over a 2-month period	±34 hours	Ongoing supervision to CHW during implementation by the director of the promotora programme	One CHW leads or runs the session, the second provides logistical support, including weighing participants
5. Katula et al., 2010 [14] 6. Blackwell et al., 2011 [15] 7. Katula et al., 2011 [16] 8. Katula et al., 2013 [17] 9. Lawlor et al., 2014 [18] [HELP-PD]	Gender: 8 Females, 2 males Mean age: 57.2 years Mean education level: -8 reported some education - all (10) completed some form of diabetes self-management education	-Recruited patients with Type 2 diabetes -experience in group leadership -well controlled HbA1c -history of healthy eating, physical activity & weight loss -evidence of potential for effective leadership (prior experience & strong interpersonal skills)	Training style/approaches: -experiential learning -didactic instruction -peer monitoring -observation Content: -study protocol -intervention philosophy, goals & procedures -weight loss (energy balance) -physical activity basics -nutrition basics -group facilitation -cognitive-behavioural principles -participant monitoring & toolbox methods -data entry	36-hour programme over the course of 6–9 weeks	-Trained & monitored by registered dieticians	-Conducted the lifestyle intervention group sessions -managed group participants -data entry of participant body weights obtained in each session
10. Islam et al., 2013 [19]	Gender: NP Mean age: NP Mean education level: NP	-Trained (at a medical school), bilingual Korean-American CHW	Content and Format: -60-hour core competency-based training 30 hours of additional training on mental health; motivational interviewing & other related topics.	-60 hours, over eight days in a three-week period -30-hour additional training Total = 90 hours	No info	-Facilitated group sessions -follow-up phone calls
11. Horowitz et al., 2011 [20] [Project HEED/HEAL]	Gender: NP Mean age: NP Mean education level: NP	NP	NP	NP	NP	Delivered classes [Trained to conduct eight, 90-minute classes (6 weekly, then two bi-weekly)]
12. Duggan et al., 2014 [21]	Gender: NP Mean age: NP Mean education level: NP	NP	Content: -diabetes education -working in the community Training Resources: -CDC’s CHW Evaluation Tool Kit -training by a local diabetes specialist	100 hours	-twice-yearly refresher courses	-recruitment -conducted sessions
13. Koniak-Griffin et al., 2015 [22]	Gender: NP Mean age: NP Mean education level: Promotoras had a high school diploma or equivalent	-Four or more years’ employment as a community health worker -either resided in or had extensive work experience in the community where the study was implemented	Content: -Orientation to the study -extensive training in the curriculum -protocol-defined content -behaviour change -human subjects’ protection -structured training activities, including 4 days focusing on delivery of modules in Su Corazón, Su Vida [*Your Heart*, *Your Life]* (conducted by a bilingual promotora trainer with extensive experience implementing the curriculum and educating promotoras) -research-specific skill sessions	100 hours	-Regular staff meetings with opportunities to discuss experiences -observations of performance in classes and home visits to verify the accuracy of content and appropriateness of counselling in the Individual Teaching and Coaching sessions	-delivered 6-month intervention
14. Satish et al., 2013 [23]	Gender: NP Mean age: NP Mean education level: NP	NP	Content: -Group facilitation -communication skills -how to set & monitor goals for lifestyle behaviours -goal setting & planning for a healthy lifestyle	2 days	-Refresher training (2days) after session 5 -peer leaders contacted before & after each session to share their experiences -face-to-face meetings with groups of peer leaders at regular intervals -ongoing support & communication between the peer leaders & the K-DPP intervention team & among peer leaders	-Deliver small group sessions -keep a record of each interaction with participants & intervention team
15. Simmons et al., 2008 [24]	Gender: NP Mean age: NP Mean education level: NP	NP	Content and Format: -Trained to deliver intervention based on social cognitive theory Maori CHW training was provided in modular form covering: -basic anatomy & physiology -communication skills -motivational interviewing -broader health issues -the content & background to the 12 messages of lifestyle intervention Training resources: *A desk file (including safety & engagement principles & intervention approach) & a toolkit supporting the 12 messages were created from existing materials or developed *de novo* & distributed in a case to each Maori CHW (included a personal digital assistant (PDA) each Maori CHW was allocated a set of scales	NP	NP	-Delivered the intervention
16. DeJoy et al., 2013 [25] FUEL Your Life	Gender: NP Mean age: NP Mean education level: NP	-Peer coaches were identified by occupational nurses	Format: -Given talking points for each lesson to use in reinforcing the self-study programme -participated in a 1-hour training session with the research team Training Resource: -received a peer health coach manual	1-hour training session	NP	-Peer health coaches -ongoing informal contact with participants -responsible for providing basic information, answering simple questions -providing encouragement & support
17. Brace et al., 2015 [26] FUEL Your Life	Gender: NP Mean age: NP Mean education level: NP	-Peer health coaches were identified by occupational nurses with the intent of representing each department/shift	Format: -Had an individual meeting with research team to discuss responsibilities Training Resource: -Received a manual	NP	NP	- Ongoing informal contact with participants -to use talking points to reinforce FYL lessons -provide social support
18. Wilson et al., 2016 [27] FUEL Your Life	Gender: NP Mean age: NP Mean education level: NP	-Respected & trusted co-workers who were also participants in the programme	NP	1-hour	Site coordinator [occupational nurse] served as a resource to health coaches	-Peer health coaches -providing basic information -answering simple questions -providing encouragement & support -referral to site coordinator & research team for complex issues
19. Ockene et al., 2012 [28]	Gender: NP Mean age: NP Mean education level: NP	-Spanish speaking - post high school education -some previous undergraduate education in nutrition -none were registered dietitians	Content: -Extensive training in delivery of the intervention protocol -nutritional & exercise aspects of intervention -theoretical background -training in motivational counselling -group management skills Format: -led by a behavioural psychologist and a senior registered dietician	NP	-Booster training sessions scheduled semi-annually	Delivered intervention/sessions
20. Islam et al., 2014 [30] [Project RICE]	Gender: NP Mean age: NP Mean education level: NP	NP	Content and [some] Format] - The CHW supervisor participated in a two-part 105-hour core competency & curriculum based training [comprehensive skills training for CHW; disease prevention & management] -the CHW supervisor was trained on the delivery of the adapted curriculum -the CHW supervisor & study staff subsequently trained three additional study CHW on the study protocol, delivery & curriculum [Some] Content: -all study staff attended approximately 30 hours of additional training on mental health, motivational interviewing/basic action planning & other related issues	Not clear	NP	Facilitated group sessions
21. Thompson et al. 2015 [31]	Gender: NP Mean age: NP Mean education level: NP	-previous training in obtaining blood pressure readings using an automatic cuff -one-time training session in diabetes and CVD -ability to speak and read Spanish -ability to attend one screening session -access to telephone	Content: -one session on screening	NP	NP	-risk screening -referral
22. Oba et al., 2011 [32]	Gender: NP Mean age: NP Mean education level: NP	-Recruited based on interest in developing a diabetes mellitus programme	NP	NP	NP	-Recruited people who were at risk for developing diabetes -ran programme -provided nutrition education -selected appropriate exercise types for fitness phase
23. Balagopal et al., 2012 [33]	Gender: NP Mean age: NP Mean education level: 60% had a college degree	-At least a high school diploma -an interest in health care & community -willingness to learn -leadership qualities -bilingual (English & Gujarati) -previous healthcare or community experience -a strong commitment to work in the community	Content: -4 weeks of structured training on an existing diabetes prevention & management curriculum -skill-based knowledge using hands-on training on vital signs, dietary intake, anthropometrics & health screening -knowledge on diabetes & its risk factors & complications -ethics & confidentiality of dealing with human subjects & survey dissemination Format: -training provided by an expert & multidisciplinary team, i.e. a registered dietician, a certified health educator, a public health practitioner, an endocrinologist, a sanitation specialist, a general practitioner & an internal medicine specialist	4 weeks	NP	-Change agents to provide health education -serve as community advocates -collect data for the study
24. O’Brien et al., 2015 [34]	Gender: NP Mean age: NP Mean education level: High school education	-Dedication to the community -natural leadership skills -worked with the investigative team for 8 years -conducted several group-based lifestyle interventions	Format: -Training from local & national diabetes experts	18 hours	-Supervision & feedback before implementing the study protocol -all sessions were attended by an author	Delivered sessions
25. Benyshek et al., 2014 [35] [The Life in BALANCE pilot study]	Gender: NP Mean age: NP Mean education level: NP	NP	Format: Centralised training that included: -required reading -instructional video tapes -observation of trained personnel -audio/videotaped practice sessions -resource core review	NP	NP	Delivered core curriculum and conducted follow-up Native lifestyle coaches
26. Philis-Tsimikas et al., 2014 [36]	Gender: NP Mean age: NP Mean education level: NP	NP	Content: In-depth standardised training in the curriculum (Dulce Mothers) & group facilitation methods: -disease content -group management dynamics -motivational interviewing skills -Health Insurance Portability & Accountability Act (HIPPA) -and other health workplace regulations Format: -trained by a lead health educator with at least 5 years’ experience in leading peer-led interventions	40 hours	Supported by a multi-disciplinary team	Teach/deliver sessions
27. Cherrington et al., 2015 [37]	Gender: NP Mean age: NP Mean education level: NP	-bilingual, bicultural -good personal communication skills -a driver’s license -a desire to work in the community	Content: -topics related to each session -the principles of Motivational Interviewing Format: -role-playing & practice sessions	NP	NP	-delivered group & individual sessions
28. Ruggiero et al., 2011 [38]	Gender: NP Mean age: NP Mean education level: NP	-delivered a 1-yr Vanguard group prior to this study	Content: -group leadership skills -diabetes self-care -behaviour change & psychosocial topics -physical activity -healthy eating	NP	Ongoing supervision & support in delivering the programme	-served as a Healthy Life Coach
29. Treadwell et al., 2010 [39]	Gender: NP Mean age: NP Mean education level: NP	NP	Content: Trained to: -to recruit participants -implement the obesity & diabetes prevention intervention -facilitate exercise & healthy lifestyle activities -connect men with primary health care providers & other supportive community resources	NP	NP	-to recruit participants -data collection -implement the obesity & diabetes prevention intervention -facilitate exercise & healthy lifestyle activities -connect men with primary healthcare providers & other supportive community resources
30. Cadzow et al., 2013 [40]	Gender: 12 females, 1 male Mean age: >35–65 years Mean education level: -Two-thirds had a 4-yr college degree -one some training/coursework beyond degree -one high school diploma -one with some college training, but not completed a formal degree	-recruited through block clubs, tenant council meetings, a community health centre & a woman’s church group -upon recruitment, prior to acceptance into training programme a Baseline survey including demographics & a list of other types of leadership activities in which they had participated	Content: -Refining Leadership Skills -Cultural Broker Skill Development & Cultural Diversity Awareness -Diabetes 101 -Health Literacy & the State of Ageing & Health in America -Methods of Cultural Health Brokering [the curriculum was approved for Continuing Professional Education credit at Buffalo State College] -conducting & evaluating community conversations	4 weeks	NP	-held five community conversations, reaching 700 community members -established eight diabetes resource libraries
31. Cadzow et al., 2014 [41]	Gender: 12 females, 1 male Mean age: -35->65 years Mean education level: at least a 4-yr degree	-community members who were active in various area community organisations were recruited	Content: -addressed skills & methods in leadership -cultural health brokering -basic diabetes knowledge -health literacy	20 hours of formal training, during one week	Met monthly with programme leaders & evaluators	Neighbourhood Health Talkers -led community conversations -established a resource library
32. Staten et al., 2005 [42] [Pasos Adelante/ “Steps Forward”]	Gender: 10 females, 1 male Mean age: NP Mean education level: NP	-Recruitment of CHW/promotoras was left to the contracted agencies	Format: -Nine of the promotoras were involved in curriculum design & received ± 6hrs. of manual training -the two additional promotoras were trained individually by other promoters with technical assistance from programme personnel -**in addition,** many promotoras attended week long training at a CHW conference -**also**, the promotoras attended a variety training on diabetes	6 hours [extra training not accounted for]	Additional training was conducted when necessary throughout the programme	Facilitated programme -Led the sessions working in pairs -provided feedback on sessions
33. Staten et al., 2012 [29] [Pasos Adelante/ “Steps Forward”]	Gender: Female (3) Mean age: NP Mean education level: NP	-Lived in community for 20–35 years -two of the three promotoras facilitated the Pasos Adelante 1-yr pilot programme in their community	Content and Format: -Su Corazon, Su Vida curriculum at a national conference	One week, half day training	Ongoing training included quarterly refresher training & local & national conferences	Facilitated sessions
34. Sosa et al., 2013 [43]	Gender: NP Mean age: NP Mean education level: NP	-Recruited from a CHW certification programme	Content: -to communicate with participants -to provide health education and support -basic nutrition and physical activity knowledge -nutrition counselling skills -nutrition education -behaviour change skills -physical activity skills -health knowledge on obesity, diabetes & CVD -healthy eating and physical activity impacts on health -ethical issues in health promotion -small group dynamics/management -presentation skills -protecting human subjects -helping participants use implementation tools -leading physical activity lessons -leading cooking demonstrations -ensuring food safety -implementing health and nutrition lessons	-30 hours training -6 weeks participation in condensed version of implementation	-weekly staff meetings to provide ongoing training & support	Delivered intervention/sessions
35. Kousar et al., 2015 [44]	Gender: NP Mean age: NP Mean education level: NP	-part of communities, i.e. hold language & cultural knowledge -have potential to make lifestyle changes -have existing qualifications/experience in the education, social welfare/health sectors from their country of origin, beyond the category of “lay” or “peer”	Content: -diabetes complications -prevention & management -Day 1: Understanding of diabetes–types of diabetes; diabetes complications; prevention; the global diabetes epidemic; the role and skills of training & education necessary to become a socially active member of community; info on health professionals related to diabetes -Day 2: Understanding diet & nutrition–barriers to healthy eating; food labels; healthy food shopping; the healthy food pyramid -Day 3 –mental health & well-being in diabetes–how to collect anthropometric indicators of diabetes risk factors–administer diabetes knowledge assessments \7 dietary recall questionnaires -Day 4 –Report day–feedback evaluation by each CHV Format: -Interactive education & training workshops -presentations, guided activities & group discussions	4 days	NA	NA
36. Coppell et al., 2009 [45]	Gender: NP Mean age: NP Mean education level: NP	NP	NP	NP	NP	-not clear -recruitment
37.Schulz et al., 2005 [46]	Gender: NP Mean age: NP Mean education level: NP	[existing skills] -experience as community organisers -personal trainers -youth leaders -caregivers	Content: -detailed information about diabetes & the role of diet & physical activity -nutrition label reading -recipe modification -strategies for working with communities to address diabetes	2 weeks	Support from project coordinator & members of steering committee	HEED advocates developed: -activities to promote healthy diets & physical activity -a weekly walking club for senior citizens -events focused on diabetes awareness & prevention -cooking demonstrations -monthly fruit & veg market
38. Cohen & Ingam, 2005 [47] 39. Teufel-Shone, Drummond & Rawiel, 2005 [48] 40.Reinschmidt et al., 2010 [49]	Gender: NP Mean age: NP Mean education level: NP	-previous experience and training in community outreach services -they were uniquely familiar with the curriculum as they were involved in its development and adaption	Content: introduction to the overall flow and curriculum format; and instruction in the use of educational materials Format: role play, practicing delivery and co-worker critique of delivery styles Resources: instructional manual provides an overview of the goals and format of the programme, a description of objectives, an outline of the activities and supplies needed for each contact point	One day		-carrying out project components -involved in the development of family curriculum component -grassroots leadership **Not part of their duties:** -participated in food demonstrations -collecting signatures for a petition to create a community park in another area -implemented the programme Adaption 1: -conducted home visits -implemented educational classes Adaption 2: -recruitment and consenting -scheduling of measurement appointments -family curriculum implementation -reminder calls -attendance documentation -follow-up support
41. Whittemore et al., 2013 [50]	Gender: NP Mean age: NP Mean education level: NP	NP	NP	-4-hour training	Ongoing supervision by nurses and PI of study	-recruitment -class set up -follow-up

*NA–Not applicable

*NP–Not Provided

## Results

### Overview of the searches

**Fig 1:**
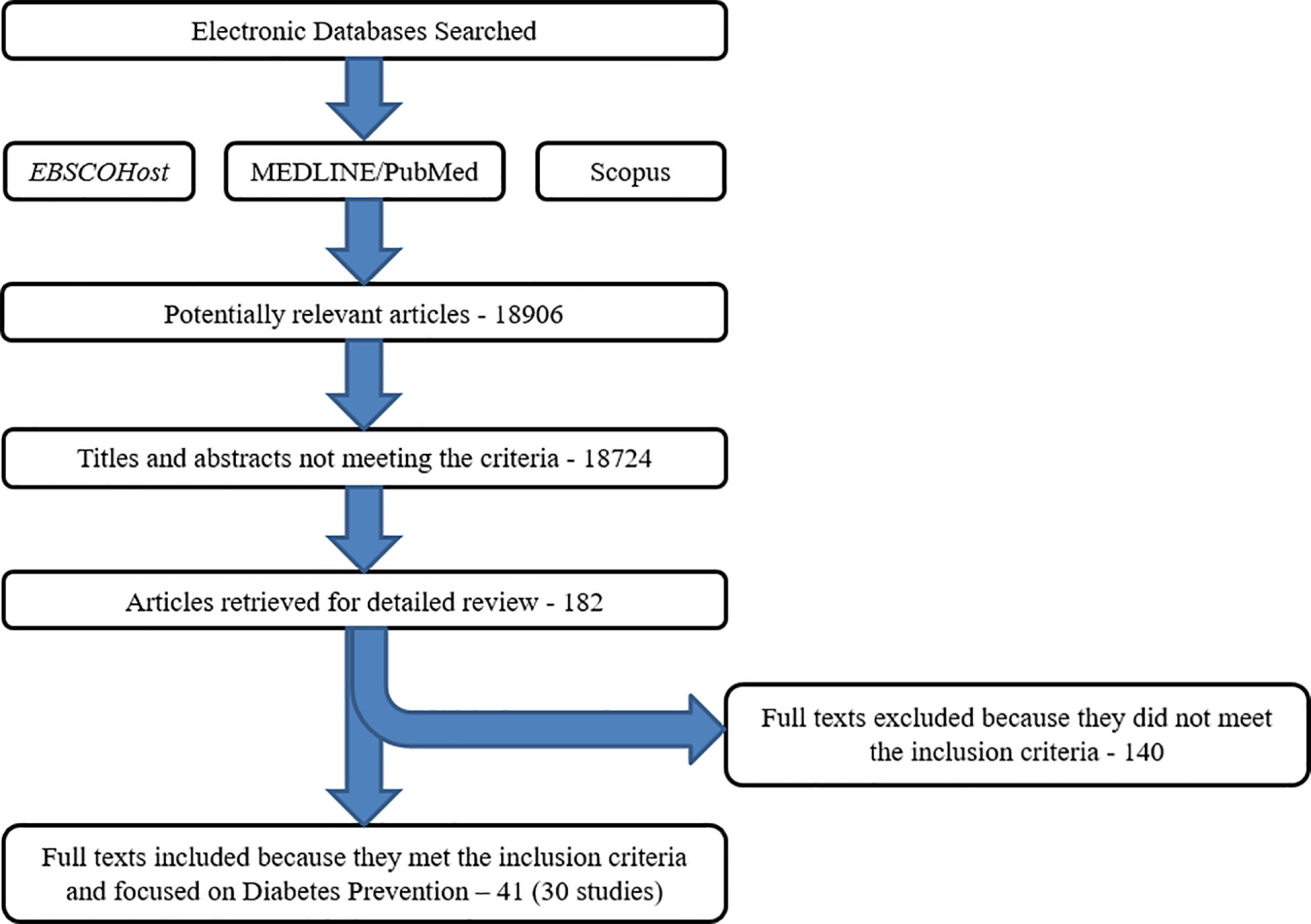
Flow diagram of retrieved studies.

Database searches identified 18906 entries; after removing duplicates and screening titles and abstracts, 182 publications were selected for further full-text evaluation (Fig 1). Forty one papers fulfilled the inclusion criteria and form the basis of this review. These 41 papers reported on 30 studies with companion papers comprising protocol papers, pilot studies, cost analyses and process evaluation papers.

### Study designs and geographic locations

Most studies (24) were conducted in the US, while two studies each were conducted in India [23, 33] and New Zealand [24, 45], and one each in Thailand [32] and Australia [44]. None of the publications originated from Africa.

The 30 primary studies included 10 RCT [10, 13, 14, 19–24, 27], 14 quasi-experimental/comparative observational studies [25, 28–39], three studies with a one-group pre- test/post-test or post-test only design [41, 43, 44], two case studies [46, 47] and a study each with an interrupted time-series prevalence survey design [45], and a mixed method embedded design [50].

### Study settings, participants and interventions

The sample sizes varied widely from 20 participants in a single community to 2369 participants in 46 communities. Intervention settings varied but were mainly community-based such as churches, homes and community centres. A study each was conducted at senior centres [10, 11] and worksites [25–27].

Studies were conducted mainly in minority populations such as Latinas/Hispanics [13, 20–22, 28, 29, 34, 36–38, 42, 43, 47] and African Americans in the US [39, 40], the Maori in New Zealand, and in people without “easy” access to healthcare services [24, 45]. Most studies (n = 22) included 18–79 year old men and women, with a female preponderance. Two studies included men only [31, 39], while six targeted women only [13, 22, 34, 36, 37, 43].

The diabetes prevention interventions and methods of delivery were culturally adapted to the target population. Most of the DPPs were lifestyle interventions; however, one study focused on diabetes education only [40, 41] and another on diabetes risk screening [31].

### Profile of community health workers

CHWs were generally from the local study community and shared the same race/ethnic and language backgrounds as the participants. Two exceptions were a worksite-specific study which made use of peer coaches [25–27] and a senior centre study of whom some of the lay health educators or coaches were staff members [12]. Nevertheless, these CHWs were likely very familiar with the communities under study. Language was a specified criteria for studies conducted in Hispanic [13, 28, 31] and Korean- American [19] communities in the US, Aborigines in Australia [44], and in Gujarat, India [33].

Most of the publications included in the review did not provide detailed information on CHW characteristics. Gender, age and education level were reported in only eight [12, 17, 29, 40–44], six [12, 17, 40, 41, 43, 44] and seven [12, 17, 22, 33, 34, 40, 41] studies, respectively. CHWs were mostly women aged between 20 and 65 years, but usually older than 35 years. Education level varied from high school education to non-health related college degrees [12, 17, 22, 33, 34] with CHWs in Buffalo City, US even having post-graduate qualifications [40, 41].

The qualifications and work experience of CHWs varied widely across studies. Qualifications included: 1) completing only high-school [13, 22, 33, 34] or 2) a CHW-certification programme [43] 3) previous training on measuring automated blood pressure (BP) [31] 4) and 5) post-high school education and some undergraduate education in nutrition [28]. Instead of qualifications, other studies focused on work experience as a CHW [22], prior or current experience in working in various areas of health [51], experience as a community organiser, personal trainer or caregiver [41, 46, 48], or having worked with the investigative team previously [34], as well as being involved in developing and adapting programme curriculum [48]. A qualification or experience in the education, social welfare or health sectors was a pre-requisite in an Australian study [44].

A single study used CHWs with well-controlled diabetes and a history of healthy eating, regular physical activity, weight loss and group leadership experience [14, 18]. In contrast, a study in Thailand recruited CHWs who were simply interested in developing a DPP [32]. Many studies emphasised soft skills in the selection of their CHWs. These included possessing leadership qualities [13, 33, 34, 40, 41], good communication skills and the ability to listen [37], and a dedication to the community [13, 34, 37].

## Community health worker training

The duration of the training varied considerably among studies, from a single one-hour session [25, 27] to 40 hours [36], and even 100 hours or more [21, 22, 30]. Overall, the training was culturally sensitive and/or appropriate (Table 2). Topics covered included the importance of good nutrition [32, 43, 44] and the benefits of increased physical activity [14, 38, 43, 46]. Communication [23, 24] and leadership [38, 40, 41] skills to run group sessions, and motivational interviewing to facilitate behaviour change [19, 24, 28, 30, 36, 37] were also emphasised. Most studies utilised interactive training and role-playing to impart skills during the training sessions. Four studies did not describe CHW training [32, 45, 47, 50]. One study put specific emphasis on intervention fidelity/following study protocol [12].

Post-training support ranged from daily supervision to monthly meetings. These included ongoing supervision, monitoring and support [13, 14, 23, 38], weekly technical conference calls [10], weekly staff meeting [43], and monthly meetings with programme leaders and evaluators [41].

Some studies also provided continuous or booster training. These included ongoing training with quarterly refresher training sessions as well as local and national conference attendances [29, 42], biannual booster training sessions [21, 28] and a two-day refresher training mid-way through the intervention programme [23].

### Community health worker responsibilities

As summarised in Table 2, most CHWs in these studies delivered the intervention activities, i.e. led or facilitated the group sessions. These included developing and organising activities to promote healthy diets and physical activity such as a weekly senior citizen walking club, diabetes awareness and prevention events, cooking demonstrations, and a monthly fruit and vegetable market [10, 11, 13–24, 28–30, 32–39, 42, 43, 47, 51]. In Buffalo City (US), CHWs led community “living diabetes well” conversations and established “diabetes resource libraries”, which provided information on diabetes, healthy living, healthcare providers, and recipe cards and cookbooks [40, 41]. CHWs linked African-American men with primary healthcare providers and other supportive community resources [39], and in migrant farmworkers screened and referred those at high risk for diabetes and cardiovascular disease (CVD) [31].

Three of the included studies, however, used occupational [27], homecare [50] and rural health nurses [45], instead of CHWs, to deliver the interventions. CHWs only recruited [45] and maintained regular contact with participants providing ongoing support and information [27], and set-up meeting venues and coordinated walking groups [50] in these studies.

CHWs also recruited programme participants in four of the studies where they led the interventions [21, 32, 39, 49] and provided follow-up support between the intervention programme/sessions in three studies [30, 35, 49]. Other CHW tasks included data collection [39] and entry [14, 17], and recording attendance [18, 49] and interactions between participants and project staff [23].

### Diabetes prevention programme outcomes

Outcomes of the DPPs are reported in Table 1. Most studies (20) included a measure of adiposity such as weight or waist circumference with weight loss ranging from more than 7% of body weight [38] to modest weight loss of 1.1kg [28]. Unexpectedly, a study reported a significant difference between groups because of weight maintenance in the intervention group, but weight gain in the control group [27].

Eleven studies reported on biochemical outcomes. Blood glucose and insulin levels were measured in 10 studies; it was the primary outcome in six of these studies [14, 18, 22, 28–30, 33–35, 37, 39, 45]. Six studies reported significant improvements in glucose levels between intervention and control groups or post- and pre-intervention periods [16, 18, 28–30, 33]. For example, greater reductions were found in the intervention vs. the usual care groups (-4.3 mg/dL vs. -0.4 mg/dL) [16]), in individuals with impaired fasting glycaemia vs. normoglycaemia (-6.02 mg/dL vs. -1.3 mg/dL) [33], and at the study conclusion vs. during follow-up (-4.529mg/dL vs. 0.686 mg/dL) [29]. Statistically significant reductions in insulin (6.5 uU/ml vs. 2.7uU/ml) and insulin resistance (HOMA-IR) (1.9 vs. 0.8) in the intervention compared to the usual care groups were reported in only a single study [16]. Glycated haemoglobin (HbA1c) was measured in four studies [21, 28, 34, 36] with improvements noted in two studies on Hispanic communities [21]. One study reported a proportionate HbA1c reduction of 37.5% vs. 0.44% in the intervention compared with the delayed intervention group [21], while the other described an absolute reduction of 0.10% vs. 0.04% in the intervention compared to the usual care group in [28].

BP was measured in 13 studies [19, 22, 28–36, 39, 45] with improvements noted in eight. Four studies reported significant improvement in both systolic BP (SBP) and diastolic BP (DBP) with reductions of 3.2 mmHg to 13 mmHg for SBP and 2.3 mmHg to 5.1 mmHg for DBP [29, 30, 33, 36]. A single study each reported a 23% decrease in overall BP level [39], a 2.61 mmHg drop in SBP [32] and a 6.2 mmHg reduction in DBP [34].

Lipid levels were measured in nine studies [22, 28–30, 34–37, 45] with improvements found in five studies. Three studies each reported significant improvements in total cholesterol [29, 36, 37] and low-density lipoprotein cholesterol levels [34, 36, 37].

Nine studies reported on behavioural changes pertaining to greater physical activity levels with different variables assessed [19, 21, 22, 30, 36, 37, 39, 42, 48]. Significant improvements in physical activity were measured as 1) increases in physical activity between baseline and six months [30], and in particular vigorous activity [37], 2) increases in moderate and vigorous walking levels [42], 3) greater aerobic exercise, flexibility and strength [36], 4) increased fitness levels [39], and 5) better social interaction and greater confidence in performing physical activities [19].

Of the nine studies that assessed various food and dietary behaviours, six reported significant improvements. These dietary variables included general food behaviour [22, 42], consumption of sweetened beverages [48], and fruit and vegetable [30]; dietary fat intake [36], and caloric intake and proportion derived from protein sources [37].

Eight studies assessed knowledge on diabetes and/or CVD with all reporting significant improvements [19, 22, 30, 39, 41, 43, 44, 48]. Improved mental health outcomes were noted in two studies; the Personal Health Questionnaire (PHQ-2) and the Generalized Anxiety Disorder Scale (GAD-2) were used in a US Korean community [19] and the Patient Health Questionnaire (PHQ-8) in a US Hispanic population [37]. A single study that examined fatalistic and cultural diabetes beliefs, measured by the Power Fatalism Inventory, showed significant reductions in fatalistic beliefs about diabetes manageability and endorsement of culturally driven diabetes beliefs [36]. Although two studies collected data on quality of life outcomes, neither study reported these findings [14, 23].

### Cost analyses of diabetes prevention programmes

Two studies, both from the US, conducted cost analyses for their DPPs [11, 18]. In the study conducted in senior centres, total estimated cost for the CHW delivered lifestyle intervention was $2731 per senior centre or $165 per participant or $45 per kilogram weight lost [11]. These costs were almost half those of a health professional delivered DPP which cost $300 per participant or $88 per kilogram lost [52]. Direct medical costs per participant in the HELP-PD lifestyle intervention programme conducted over two years were $850 compared to $2361 for the first two years in the original DPP conducted in the US [18].

### The effect of community health workers on programme outcome

Studies did not specifically compare the use of CHWs vs. health professionals on programme outcomes, except for Thompson et al. [31] who found that CHWs perform as well as registered nurses in the use of non-invasive risk screening tools. Therefore, it is difficult to quantify the specific advantages of using CHWs to implement DPPs. However, DPPs that targeted vulnerable/underserved communities highlighted the importance of CHWs in contributing to their acceptability and appropriateness (Table 3) [21, 22, 24, 30, 35]. Additionally, studies have also emphasised the importance of community participation per se as a major contributor to programme effectiveness [32, 33, 38, 46–49]. The shared culture and language between CHWs and participants likely contributed to better programme implementation and outcomes [19, 21, 30, 36, 44]. In essence, using CHWs in DPPs that target culturally and linguistically diverse groups seem to be a credible strategy [44].

**Table 3:** Direct CHW effect/contribution as recognised by study authors.

Study	CHW impact
West et al., 2011 [10]	“Lay health workers offers a promising vehicle for translation of evidenced-based obesity treatment in underserved areas”
Krukowski et al., 2013 [11] Krukowski et al., 2013 [12]	“A LHE [lay health educator] DPP translation in senior centers is effective in achieving weight loss at low cost and offers promise for the dissemination of this evidenced-based intervention” “Training LHE’s to disseminate evidenced-based interventions holds great promise for improving public health and access to effective care, particularly in medically underserved areas”
Katula et al., 2013 [17]	“A DPP administered through an existing community-based system and delivered by CHWs is effective at inducing significant long-term reductions in metabolic indicators and adiposity”
Islam et al., 2013 [19]	“…CHWs can facilitate support by serving as a bridge to the health care system and proving culturally- and linguistically- tailored health education”. …many participants felt connected to, and appreciative of the CHWs’ efforts, suggesting that CHWs serve a unique role in health promotion effort”
Duggan et al., 2014 [21]	“Bilingual CHWs removed linguistic barriers”
Koniak-Griffin et al., 2014 [22]	“Findings of this RCT support the feasibility and positive outcomes of implementing a promotora-facilitated Life Behaviour intervention in the community with overweight/obese Latinas”
Simmons et al., 2008 [24]	“Intensively treated participants would have had one or more unrecorded MCHW [Maori CHW] encounters and these may have had a significant effect” “The MCHW intervention was seen as key to the downstream intervention activities”
Staten et al., 2005 [42]	“The Pasos Adelante Program has demonstrated that an educational curriculum in conjunction with the support of promotoras can motivate people to healthy lifestyle behaviors”
Islam et al., 2014 [30]	“Participants reported positive feedback about the program and about the CHWs, particularly regarding the cultural congruence of CHWs and the strong connectedness of the intervention with community resources and cultural and religious norms and values”
Oba et al., 2011 [32]	“The research found that the health volunteers in each community were important for conducting the health promotion activities in their own villages”
Balagopal et al., 2012 [33]	“…that CHWs can provide advice on lifestyle modification and improve awareness of diabetes and CVD similar to allied health professionals in earlier studies… The CHWs were able to successfully empower the women to speak up at their meetings and instil them with confidence in their decision making abilities as related to their health and well-being. Using CHWs strengthened the links among project personnel, the community and existing community networks”
Philis-Tsimikas et al., 2014 [36]	“Participants appreciated the convenient community location, social support received from other participants and the promotoras”
Cherrington et al., 2015 [37]	“…peer delivered interventions may be particularly well-suited to the needs of immigrant women in newly emerging Latino communities”
Ruggiero et al., 2011 [38]	“For this project, the training, quality, professionalism and commitment of the CHWs that worked with program participants had an instrumental impact on high rates of retention and engagement of participants with project activities”
Sosa et al., 2013 [43]	“…evaluation results showed that promotoras were capable to deliver and retain participants in a lifestyle intervention program”
Kousar et al., 2015 [44]	“Here we confirm that CHV [community health volunteers] represent an effective tool for health promotion within culturally and linguistically diverse communities and have the capacity to incorporate evidence-based collection as part of their health education”
Cadzow et al., 2013 [40]	“Participants not only developed proficiency in discussing this important issue with their neighbors and peers, they also made important lifestyle changes that demonstrated their commitment to the cause and project” “The Neighborhood Health Talkers’ commitment to the program and embeddedness in their own communities resulted in an unpredicted reach to community residence”
Thompson et al., 2014 [31]	“This quasi-experimental study supports the hypothesis that Latino CHWs can use non-invasive diabetes and CVD screening tools with similar accuracy as a registered nurse”
Coppell et al., 2009 [45]	“A key factor underpinning the program was early involvement of CHWs, community members and local organizations and the use of local resources and talents so that the program would become embedded into everyday community life and sustainable in the long term.
Cohen & Ingram, 2005 [47]	“The curriculum for the family component, for example, was developed collaboratively between academics and promotoras who had never worked together in the past”
Whittemore et al., 2014 [50]	“…better understanding of the role of the CHW in the delivery of health programs is needed”

## Discussion

The current review provides evidence from 33 studies of the increasing involvement of CHWs in implementing DPPs. The majority of the studies, however, were undertaken in high income countries, particularly the US. Only three studies were conducted in developing countries, where the incorporation of CHWs in resource-limited settings is likely to have a greater impact.

Most studies targeted the underserved minority such as African-American and Hispanic communities in the US, Aborigines in Australia and the Maori people in New Zealand [24]. The same was true of developing regions where rural communities were the focus in India [23, 33] and Thailand [32]. DPPs that target minority and other vulnerable groups are likely suited to use CHWs who share a common culture, belief system (tradition), and language with the programme participants. This accords with The Centers for Disease Control and Prevention’s Policy Brief (2015) which produce strong evidence for the use of trained lay people i.e. CHWs as a best practice for reducing CVD risk and improving outcomes in high-risk minority populations [53]. Unfortunately, the existing evidence is unclear, inconsistent and insufficient to inform the scaling up of DPPs in diverse settings using CHWs. The complexity of the programmes precluded attributing any specific benefit to the use of CHWs. Similar to these findings, Shah and colleagues noted that despite the large body of literature on CHWs and diabetes care, the wide range of CHW roles and differing outcomes made it difficult to draw conclusions on their overall effectiveness [5].

The outcomes of interest in the included studies were mostly intermediate, such as changes in behaviour or body weight, with no study reporting diabetes incidence. Nevertheless, CHWs are regarded as adding value to programmes by fulfilling a gap/an unmet need [54]. CHW-led interventions are optimally suited to programmes where there is greater community involvement, as shown by a number of studies in this review, which used community-based participatory research approaches [19, 20, 22, 30, 32, 33, 35, 37, 38, 46–48].

The profile, scope of roles played, and training received by CHWs varied substantially across programmes. Most DPPs provided information on the training/curriculum to varying degrees [32, 45, 47, 50]. The duration of training varied significantly with shorter training sessions generally scheduled in programmes with fewer CHW responsibilities. While a systematic review reported that the performance of CHWs might improve with regular supervision and continuous training, an optimal model was not suggested [55]. Additionally, CHWs would benefit from clearly defined roles and clear processes for communication [55]. Guidelines that describe the potential roles of CHWs, the appropriate training required for each task/role and the type of programmes that would benefit from CHW involvement may be useful. While such guidelines may need to be adapted for different populations, they may encourage greater utilisation of CHWs in in appropriate healthcare programmes, including DPPs, and lead to better outcomes.

The frequency and duration of the DPP interventions varied, with higher versus lower intensity programmes having the greater impact. For example, CHWs in the HELP-PD programme delivered the intervention sessions, which comprised weekly group sessions for six months, and a monthly session for the following 18 months [16]. This study achieved significant improvements in blood glucose and insulin levels, insulin resistance, weight and BMI with the effects sustained over a two-year period [15–17]. Identifying the ideal programme intensity and duration may be useful/of value for optimal outcomes; however, this may possibly vary depending on the outcomes targeted. In addition, longer-term studies are required to ascertain the need to provide intermittent support beyond the 2-year period, which was the maximum duration of the studies included in this review.

The high concentration of studies from developed countries, especially the US, limits the generalisability of our findings. However, most populations studied were underserved or minority communities, which may be of relevance to developing region settings. The inclusion of only published studies in English language in this review may have missed lessons being learnt from potential ongoing CHW-led DPPs, particularly from developing regions, or studies published in other languages. Additional information was not obtained from the study authors to provide greater insight on CHW education, training, experience and supervision and attrition rates. Therefore, the potential for publication bias is evident in this review.

## Conclusions

In view of the labour-intensive nature of community-based healthcare programmes, particularly DPPs, and the high cost of professional healthcare staff, pragmatic cost-effective solutions are required for optimal outcomes. The utilisation of adequately trained CHWs with ongoing supervision to perform clearly defined tasks is likely to be most beneficial, as noted in a World Health Organization report on the state of evidence of CHW programmes, “…they must be carefully selected, appropriately trained and–very important–adequately and continuously supported” [3]. In addition to the aforementioned, the success of CHW-led programmes lies in the commonalities such as language, culture and tradition shared between the CHWs and participants. These commonalities facilitate communication and dialogue, which is crucial, and may play a key role in the success of such programmes. However, considering the complexity of DPPs and the diverse roles played by CHWs, it is difficult to disentangle the specific contribution of CHWs to these programmes. Nevertheless, developing guidelines for potential CHW roles and determining the appropriate level of training required may help identify the optimal CHW contribution to DPPs, which is currently lacking in the literature. Furthermore, this may perhaps encourage the wider uptake of CHW-led DPPs and lead to the development of better programmes.

## Supporting information

S1 Checklist PRISMA 2009 checklist. (DOCX)
